# Heat shock pretreatment of mesenchymal stem cells for inhibiting the apoptosis of ovarian granulosa cells enhanced the repair effect on chemotherapy-induced premature ovarian failure

**DOI:** 10.1186/s13287-018-0964-4

**Published:** 2018-09-26

**Authors:** Xiaoying Chen, Qing Wang, Xinran Li, Qingru Wang, Jiaxin Xie, Xiafei Fu

**Affiliations:** Department of Obstetrics and Gynecology, Zhujiang Hospital, Southern Medical University, Guangzhou, Guangdong People’s Republic of China

**Keywords:** Heat shock, Bone marrow-derived mesenchymal stem cells, Premature ovarian failure, Chemotherapy

## Abstract

**Background:**

Premature ovarian failure (POF) is a severe complication associated with chemotherapy for female patients of childbearing age. A previous study has shown that bone marrow-derived mesenchymal stem cells (MSCs) can partially repair the damaged ovarian structure and function following chemotherapy. Heat shock (HS) is a pretreatment to enhance cell survival. The present study aimed to demonstrate the repair effect and potential working mechanism of HS MSCs on chemotherapy-induced POF.

**Methods:**

Rat MSCs were isolated, cultured and identified. At 24 h, 48 h and 72 h after different strengths of HS pretreatment for 30 min, 1 h, 2 h and 3 h, apoptosis of MSCs was detected to determine the optimal conditions. Apoptosis and cell proliferation changes of MSCs were detected under the optimal conditions of HS. Apoptosis of HS preconditioned MSCs was detected after adding phosphamide mustard (PM) to mimic the microenvironment under chemotherapy. Rat granulosa cells (GCs) were isolated and cultured. PM was added and apoptosis of GCs was detected after coculture with the pretreated MSCs. The rat model of chemotherapy-induced POF was established and the pretreated MSCs were injected into bilateral ovaries. Ovarian structure and endocrine function were evaluated by ovary weight, follicle count, estrous cycle and sex hormone levels. Apoptosis of GCs was detected by TUNEL assay.

**Results:**

The apoptosis rate of MSCs with 1 h of HS pretreatment decreased significantly, so 1 h was considered the optimal duration. Under this condition, the reduction in the apoptosis rate persisted until 120 h after the pretreatment and cell proliferation was accelerated. After HS pretreatment, MSCs displayed an increased tolerance to microenvironment under chemotherapy. After coculture with the HS-pretreated MSCs, PM-induced apoptosis of GCs decreased. Injection of the pretreated MSCs into the rat ovaries caused an increase in ovary weight and the number of follicles at different stages of estradiol levels, and a decrease in follicle stimulating hormone levels and apoptosis of GCs in the POF model.

**Conclusion:**

HS pretreatment enhanced the repair effect of MSCs on chemotherapy-induced POF. The reason for this may be the further vitality enhancement of MSCs, which led to a greater inhibition of apoptosis of GCs.

## Background

Chemotherapy is now widely used to treat cancer and immune diseases. In recent years, the incidence of cancer has been increasing in younger adults, and more young women are affected by immune diseases and receive chemotherapy. Although survival can be prolonged by chemotherapy, some side effects occur [1], among which chemotherapy-induced premature ovarian failure (POF) has drawn increasing attention. Low estrogen levels and infertility are the two major threats to women of childbearing age [2]. However, none of the existing treatments can provide a radical cure.

Recent development in stem cell transplantation has opened up a new prospect or treatment for chemotherapy-induced POF. Animal experiments have indicated that mesenchymal stem cell (MSC) transplantation can partially reverse ovarian damage caused by chemotherapy [3]. However, the treatment efficacy falls short of expectation, probably due to the loss of transplanted MSCs. Therefore, how to increase survival of the transplanted cells is the key for achieving good efficacy. Heat shock (HS) pretreatment is an effective method for enhancing anti-apoptotic capacity of cells. It has been reported that HS pretreatment does reduce apoptosis of stem cells and increase cell vitality. However, no research studies have been conducted on HS pretreatment for chemotherapy-induced POF.

Four durations of HS pretreatment of MSCs were experimented. The optimal duration was determined based on the detection of apoptosis rates, and the biological features of MSCs under the optimal pretreatment duration were evaluated. Tolerance of the pretreated MSCs to the local microenvironment under chemotherapy was measured in vitro. The inhibitory effect of the pretreated MSCs on chemotherapy-induced apoptosis of GCs was also evaluated. We established the rat model of chemotherapy-induced POF, which was subjected to HS preconditioned MSC transplantation. Ovarian structure, endocrine function and GC apoptosis were detected in rats. The repair effect of the pretreated MSCs on the damaged ovarian structure and function was determined, and the potential working mechanism was discussed.

## Methods

### Laboratory animals

This study was performed in strict accordance with the recommendations in the Guide for the Care and Use of Laboratory Animals of the National Institutes of Health. The protocol was approved by the Committee on the Ethics of Animal Experiments of Southern Medical University (Permit Number: L2016039). All surgery was performed under sodium pentobarbital anesthesia, and every effort was made to minimize suffering.

Female clean-grade Wistar rats each weighing 180–200 g were provided by the Laboratory Animal Center of Southern Medical University. The rats were bred under conditions of room temperature (23 ± 2 °C), humidity 45–55% and a 12-h/12-h light–dark cycle, with free access to water. The rats were acclimatized for 3–5 days. Before formal experiments, vaginal smears were prepared and those with a normal estrous cycle were included. Baseline sex hormone (E_2_ and FSH) levels were determined by collecting 1 ml of blood samples from the tail vein.

### Isolation and culture of rat MSCs

Bilateral femurs and tibiae were harvested under aseptic conditions. The bone marrow cavity was repeatedly flushed with complete culture medium containing 10% fetal bovine serum until it turned white. The resulting cell suspension was inoculated into the T-25-cm^2^ culture flask, which was cultured at 37 °C in a 5% CO_2_ incubator. After the cells were adherent 48 h later, one half of the culture medium was replaced. Later, all of the culture medium was replaced every 2–3 days. Passage was performed in a 1:2 proportion after the cells grew to about 80% confluency. The cells were digested with 0.25% trypsin and passaged at a cell density of 5 × 10^4^ cells/ml. Rat MSCs of the third generation were harvested and made into a single cell suspension. Surface markers CD44, CD45, CD29 and CD34 were detected by flow cytometry. Rat MSCs of the third generation in a good growth status were harvested for subsequent experiments.

### Isolation and culture of rat GCs

Pregnant mare serum gonadotropin (PMSG) was subcutaneously injected into the female Wistar rats aged 3–4 weeks at 60 IU/rat. The rats were sacrificed 48 h later and the ovaries were harvested. Mature follicles were pricked with a syringe needle under the anatomic microscope to release GCs into the culture medium. The cells were washed with PBS twice and resuspended in an appropriate amount of culture medium for 24 h.

### Determining the optimal duration of HS pretreatment

Four durations of HS pretreatment were set up, namely, 30 min, 1 h, 2 h and 3 h at 42 °C. Five groups were set up, namely, normal group, 30-min group, 1-h group, 2-h group and 3-h group. For the last four groups, the culture flasks inoculated with MSCs were sealed and placed into a 42 °C water bath for 30 min, 1 h, 2 h and 3 h, respectively. After taking the flasks out of the water bath, the culture medium was immediately changed. The cells were then inoculated at 37 °C in a 5% CO_2_ incubator for 24 h, 48 h and 72 h, respectively. The apoptosis of MSCs was detected with Annexin-V/PI. Four samples were prepared for each group at each time point.

### Changes of biological features of MSCs under optimal conditions

Two groups were set up, namely, MSCs group and HS group. After HS pretreatment of MSCs under the optimal conditions, the cells were incubated at 37 °C for 72 h, 96 h, 120 h, 180 h and 240 h, respectively. The apoptosis of MSCs was detected with Annexin-V/PI using a flow cytometer. After culture at 37 °C for 48 h, the cell cycle was detected by PI staining. Cell proliferative capacity was measured by CCK-8 assay. The cells were then inoculated into the 96-well plate at a density of 1 × 10^4^ cells/ml and a volume of 100 μl per well. For each group, the cells were inoculated to five wells. Cells of the five wells were harvested at the same time each day after HS pretreatment under the optimal conditions. The cells were incubated with 10 μl CCK-8 for 4 h, and the absorbance at 490 nm (D490) was measured using an automatic microplate reader for 7 days consecutively. The growth curves were plotted.

### Tolerance of HS-pretreated MSCs to the local microenvironment under chemotherapy

Phosphamide mustard (PM) is the metabolic product of cyclophosphamide (CTX) in vivo and has a cytotoxic effect on ovaries. Here, we applied PM rather than CTX to the in-vitro experiment.

The cells were divided into two groups, namely, HS group (HS pretreatment of MSCs under the optimal conditions) and MSCs group (no pretreatment). Into the culture medium 30 μmol/L PM was added to mimic the local ovarian microenvironment under chemotherapy. The apoptosis rate of MSCs was detected 24 h later using a flow cytometer, and the tolerance was assessed.

### The effect of HS pretreatment of MSCs on chemotherapy-induced apoptosis of GCs

Four groups were set up, namely, normal group, PM group, MSCs group and HS group. No PM was added to the GC culture in the normal group; 30 μmol/L PM was added into the PM group to induce apoptosis of GCs; PM was added to the MSCs group and HS group to induce apoptosis, and 24 h later the GCs were cocultured with MSCs and HS-pretreated MSCs in a 1:1 ratio. Apoptosis of the GCs was detected with a flow cytometer 48 h later.

### Establishment of the chemotherapy-induced POF model in rats

Chemotherapy-induced POF was induced in rats by intraperitoneal injection of CTX. After the initial CTX dose of 50 mg/kg, intraperitoneal injection was performed at a dose of 8 mg/kg for 14 days [3, 4].

### Labeling the HS-pretreated MSCs with CM-Dil

MSCs were prepared into a cell suspension after HS pretreatment for 48 h. An appropriate amount of 1 mg/ml stock solution of CM-Dil was added at a final concentration of 4 μg/ml. After incubation at 37 °C in an incubator for 30 min, the cells were centrifuged at 1000 r/min for 5 min. The supernatant was discarded and washed with PBS twice.

### Cell transplantation and posttransplantation observation

The rats were randomly divided into five groups, with 25 rats in each group, namely, normal group, model group, sham group, MSCs group and HS group. Chemotherapy-induced POF was induced for the last four groups. At day 1 after modeling finished, the sham group received injection of 20 μl of normal saline into each ovary; MSCs group received injection of 20 μl cell suspension containing 1 × l0^6^ MSCs into each ovary; the HS group received injection of 20 μl of cell suspension containing 1 × l0^6^ HS-pretreated MSCs; for the MSCs group and HS group, the cells were labeled with CM-Dil before transplantation.

Estrous cycle was detected by preparing vaginal smears at 8:00 am each day after transplantation. Sex hormone levels (E_2_ and FSH) were detected in the serum from all rats during the estrous cycle at day 1, day 15, day 30, day 45 and day 60 after transplantation, respectively. At each time point in each group, five rats were randomly selected for sacrifice. Ovaries were harvested, and ovary weight, ovarian structure, follicle count and apoptosis of GCs were detected.

### Detection of E_2_ and FSH levels

The FSH concentration was determined with the FSH radioimmunoassay kit (Beijing Beifan Biotechnology Co., Ltd). The E_2_ concentration was measured by chemiluminescence method.

### Ovary weight, follicle count and ovarian morphological analysis

Ovaries were harvested after the rats were sacrificed, and the adipose tissues were removed. The ovaries were weighed and the gross ovarian structure was observed with naked eyes. The tissues were paraffin embedded, sectioned into 5 μm slices and subjected to HE staining. Ovarian structure was observed under the microscope. Follicles were counted using the method described in the literature [5]. Every twelfth slice was chosen to count the follicles at different developmental stages. Primordial follicles only had a single layer of fusiform GCs; in the primary follicles at least three single-layer GCs were columnar in shape; secondary follicles had at least two layers of GCs; and antral follicles had at least two layers of GCs and also a follicular antrum.

### Detection of GC apoptosis

The apoptosis of GCs was detected using TUNEL assay according to the instruction manual. The stained apoptotic cells were counted in every 100 GCs at eight positions under the microscope. The proportion of apoptotic GCs was calculated.

### Statistical process

All statistical analyses were undertaken using SPSS 20.0 software. Measurements obeying the normal distribution were expressed as $\overline{x}\pm s$. Multiple comparisons were performed by using one-way ANOVA. Pairwise comparisons were conducted using the SNK-q test or Dunnett’s T3 test. *P* < 0.05 indicated significant difference. Multiple comparisons of row × column data were conducted using Fisher’s exact test, and pairwise comparisons by chi-square test. The significance level was set as α = 0.0045 after correction.

## Results

### Culture, identification and labeling of MSCs

**Fig. 1:**
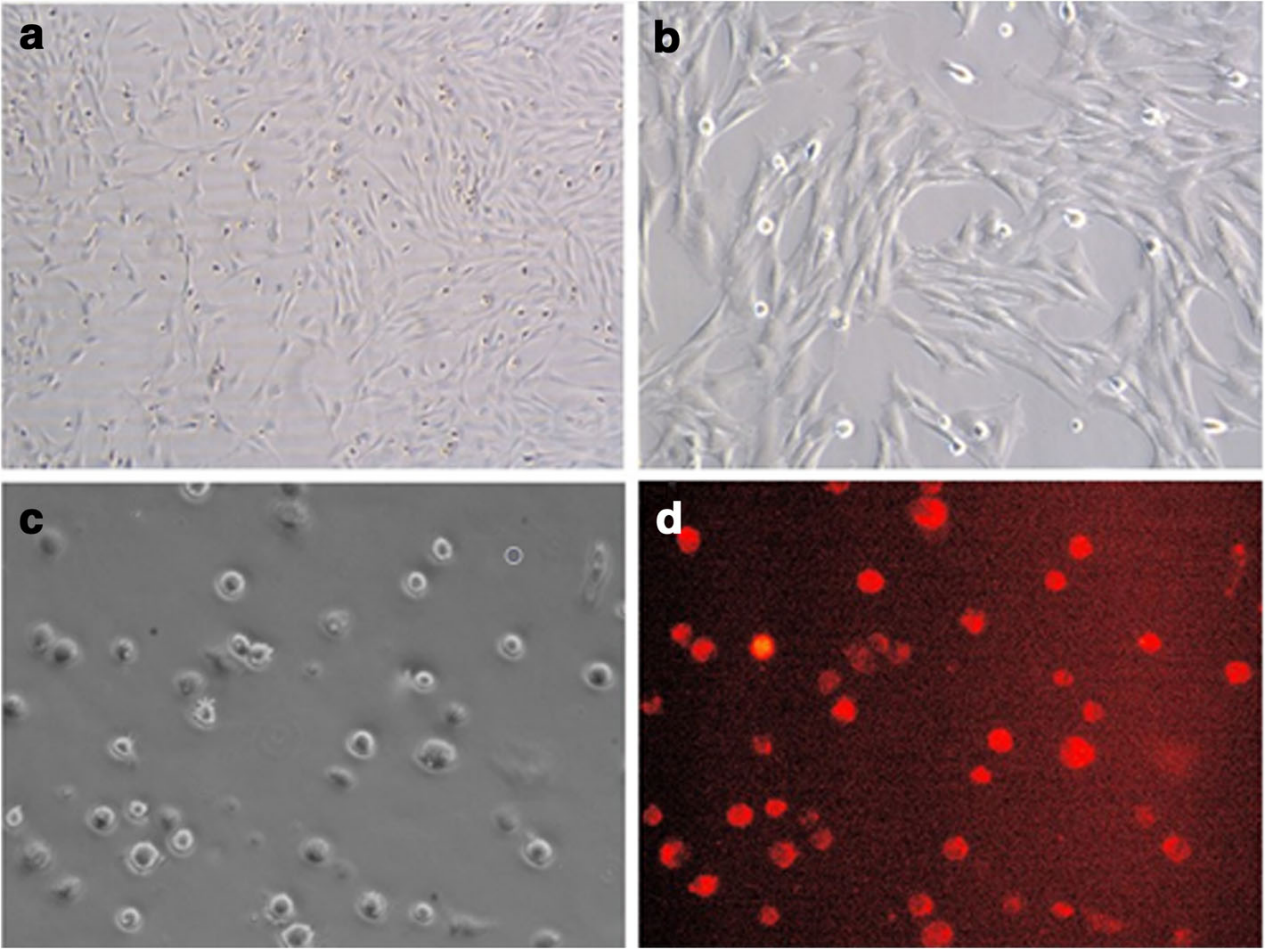
Rat bone marrow mesenchymal stem cells and CM-Dil-labeled MSCs. **a** Primary MSCs at 6 days (white light ×50). **b** Primary MSCs at 6 days (white light ×100). **c** Suspended MSCs labeled with 4 μg/ml CM-Dil (white light × 200). **d** Suspended MSCs labeled with 4 μg/ml CM-Dil (fluorescence ×200)

Round suspending cells of uniform size were observed immediately after inoculation. Cells became adherent at 24 h post inoculation. More adherent cells were observed at 72 h, presenting a fusiform shape, colony-like growth, poor refractivity and lack of three-dimensionality. At 6–7 days of culture, there were more colonies merging into patches. The first passage was performed at 80% cell confluency (Fig. 1a). After three or four passages, cell morphology became uniform as a fusiform shape and the cell arrangement was regular. Flow cytometry indicated that over 90% of the cells did not express CD34 and CD45, but positively expressed CD44 and CD29, consistent with the previous report. So, the cultured cells were MSCs and not hematopoietic stem cells. CM-Dil-labeled cells emitted red fluorescence under green fluorescence excitation (Fig. 1d) and displayed identical morphology to the nonstained cells. The labeling rate was 96.13% ± 0.52%.

### Optimal conditions of heat shock pretreatment for MSCs

**Fig. 2:**
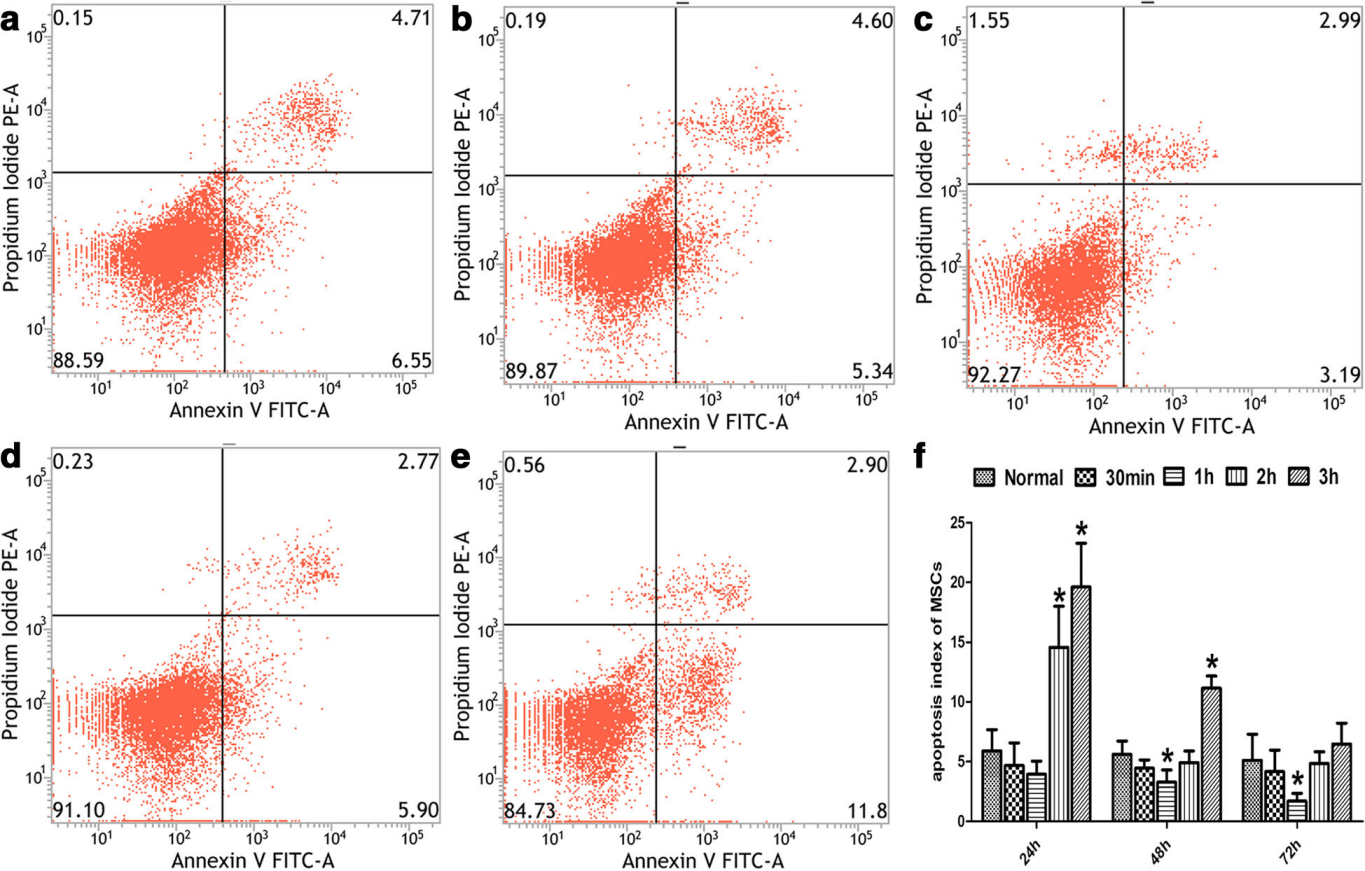
Optimal condition of heat shock pretreatment for MSCs. **a**–**e** Apoptosis of MSCs at 48 h after HS pretreatment detected by flow cytometry: **a** normal group, **b** 30-min group, **c** 1-h group; **d** 2-h group; **e** 3-h group. **f** Apoptosis of MSCs at 24 h, 48 h and 72 h after HS pretreatment. *P < 0.05, compared with normal group. MSC mesenchymal stem cell

The apoptosis rate of MSCs pretreated by HS for different durations (0 min, 30 min, 1 h, 2 h, 3 h) was measured by flow cytometry. One-way ANOVA was used for intergroup comparisons, which revealed significant difference (*F*_24 h_ = 29.408, *P*_24 h_ = 0.000; *F*_48 h_ = 39.206, *P*_48 h_ = 0.000; *F*_72 h_ = 4.884, *P*_72 h_ = 0.010). The apoptosis rates at 24 h after HS pretreatment were 5.88% ± 1.80%, 4.67% ± 1.91%, 3.93% ± 1.11%, 14.58% ± 3.42% and 19.61% ± 3.66% for the normal group, 30 min group, 1 h group, 2 h group and 3 h group, respectively. The apoptosis rate was the lowest in the 1 h group. At 48 h and 72 h after HS pretreatment, the apoptosis rates decreased in all four groups, with the lowest observed in the 1 h group (Fig. 2). Therefore, the optimal conditions were 42 °C for 1 h and were used for all subsequent experiments.

### The effect of HS pretreatment on the biological features of MSCs

**Fig. 3:**
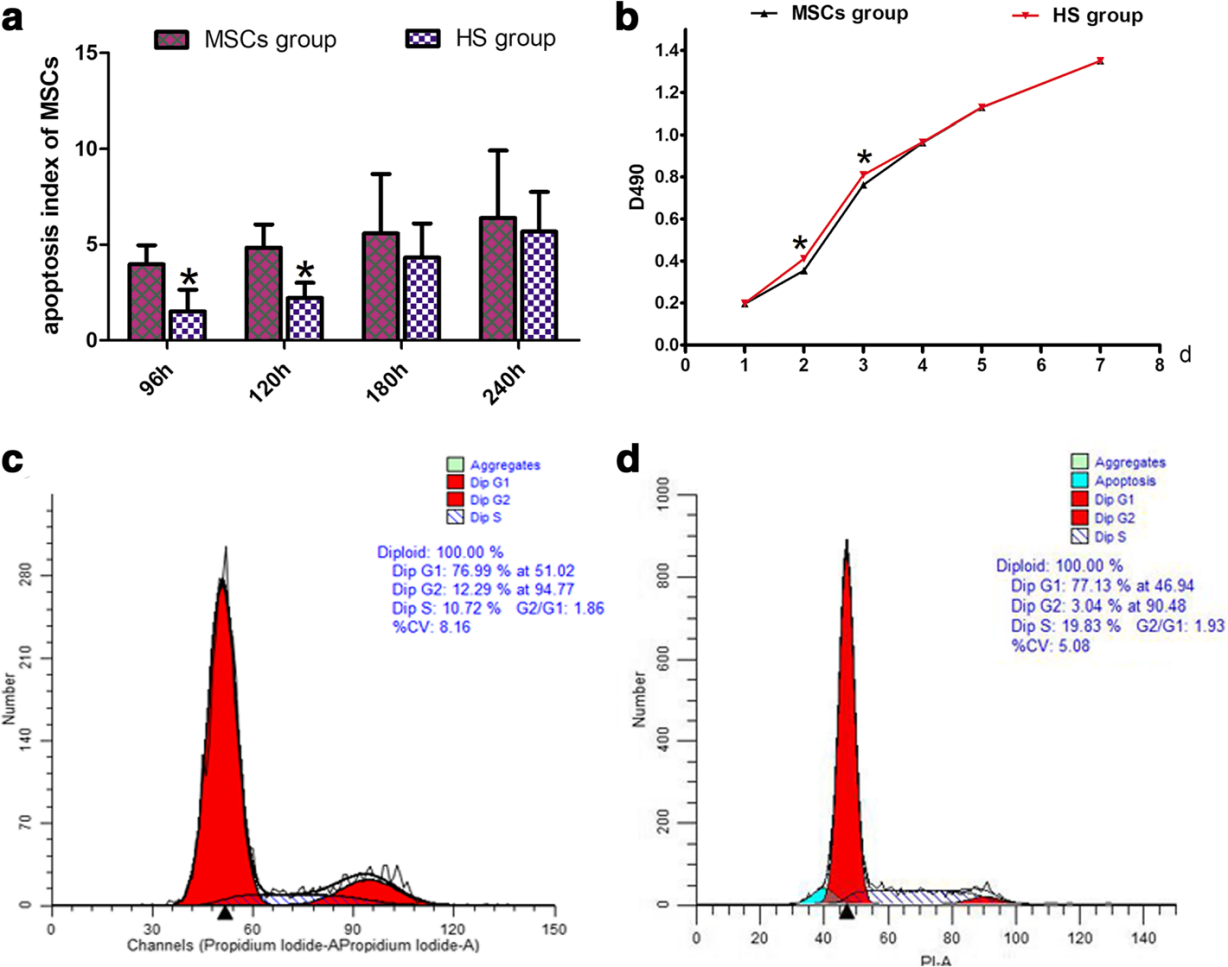
Effect of heat shock preconditioning on biological characteristics of MSCs. **a** Apoptosis rate of MSCs at 96 h, 120 h, 180 h and 240 h after HS at 42 °C for 1 h. Detected proliferative ability of MSCs with CCK-8. **b** Growth curve of HS MSCs and untreated MSCs. Flow cytometry to detect cell cycle by propidium iodide (PI) method at 48 h after HS. **c** Representative data for normal MSCs. **d** Representative data for HS MSCs. *P < 0.05, compared with normal group. HS heat shock, MSC mesenchymal stem cell

Flow cytometry was used to detect the apoptosis rates of the pretreated MSCs at 96 h, 120 h, 180 h and 240 h, which were 1.51% ± 1.13%, 2.21% ± 0.79%, 4.32% ± 1.79% and 5.68% ± 2.07%, respectively. Thus, the apoptosis rates of MSCs at 96 h and 120 h post pretreatment were significantly lower than those of the nonpretreated MSCs (3.97% ± 1.00% and 4.85% ± 1.20%; *t*_96 h_ = 3.279, *P*_96 h_ = 0.017; *t*_120 h_ = 3.679, *P*_120 h_ = 0.010). However, at 180 h and 240 h the apoptosis rates of MSCs (4.32% ± 1.79% and 5.68% ± 2.07%) were not significantly different from those of the nonpretreated MSCs (5.58% ± 3.12% and 6.39% ± 3.53%; *t*_180 h_ = 0.701, *P*_180 h_ = 0.510; *t*_240 h_ = 0.344, *P*_240 h_ = 0.742; Fig. 3a). This indicated that after HS pretreatment, the anti-apoptotic capacity of MSCs was enhanced and this effect continued until 120 h post pretreatment.

Proliferative capacity of MSCs was detected by CCK-8 assay. It was found that cell proliferation was accelerated at day 2 and day 3 after pretreatment (*t*_d2_ = − 29.198, *P*_d2_ = 0.000; *t*_d3_ = − 33.686, *P*_d3_ = 0.000), indicating the promoting effect of HS on the proliferation of MSCs (Fig. 3b).

Cell cycle detection showed that the percentage of cells arrested in the S phase in the HS group was significantly higher as compared with the MSCs group (*t* = − 3.315, *P* = 0.030; Fig. 3c,d). However, there was no significant difference in the percentage of cells arrested in the G1 and G2 phases (*t*_G1_ = − 0.296, *P*_G1_ = 0.782; *t*_G2_ = 1.275, *P*_G2_ = 0.271; Fig. 3c,d).

### Apoptosis of pretreated MSCs in local microenvironment under chemotherapy

**Fig. 4:**
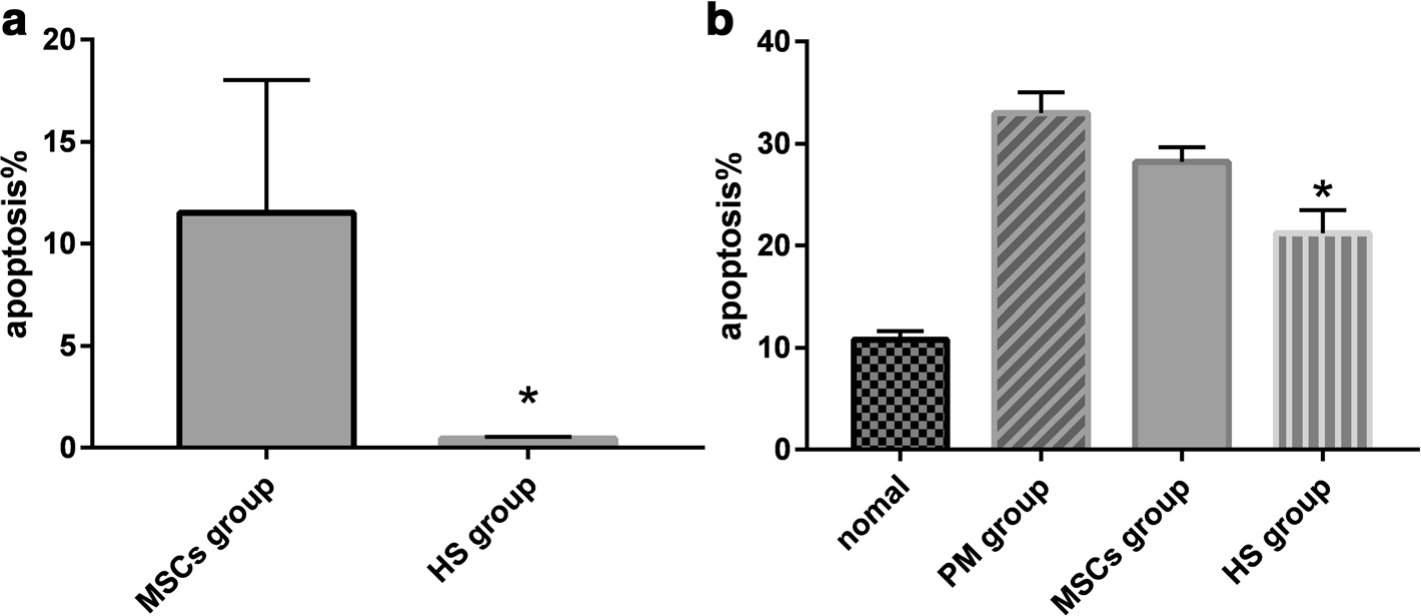
Apoptosis of MSCs and ovarian granulosa cells in each group. **a** Apoptosis of pretreated MSCs in local microenvironment under chemotherapy by flow cytometry. HS group. **b** Apoptosis rates of GCs in normal group, PM group, MSCs group and HS group by flow cytometry. HS group. *P < 0.05, compared with MSCs group. HS heat shock, MSC mesenchymal stem cell, PM phosphamide mustard

Into the culture medium 30 μmol/L PM was added to induce apoptosis. The apoptosis rate of the HS group was 0.46% and that of the MSCs group was 11.52%; it was lower in the HS group than in the MSCs group (Fig. 4).

### HS pretreatment of MSCs inhibited chemotherapy-induced apoptosis of GCs

Flow cytometry found that the apoptosis rates of the normal group, PM group, MSCs group and HS group were 10.8% ± 0.83%, 33.0% ± 2.00%, 28.2% ± 1.48% and 21.2% ± 2.28%, respectively. Statistical analysis indicated significant difference in the apoptosis rate between the four groups (*F* = 153.59, *P* = 0.00). The apoptosis rate of the HS group was lower than that of the MSCs group, but still higher compared to the normal group (Fig. 4).

### Appearance and weight of ovaries and follicle count

**Fig. 5:**
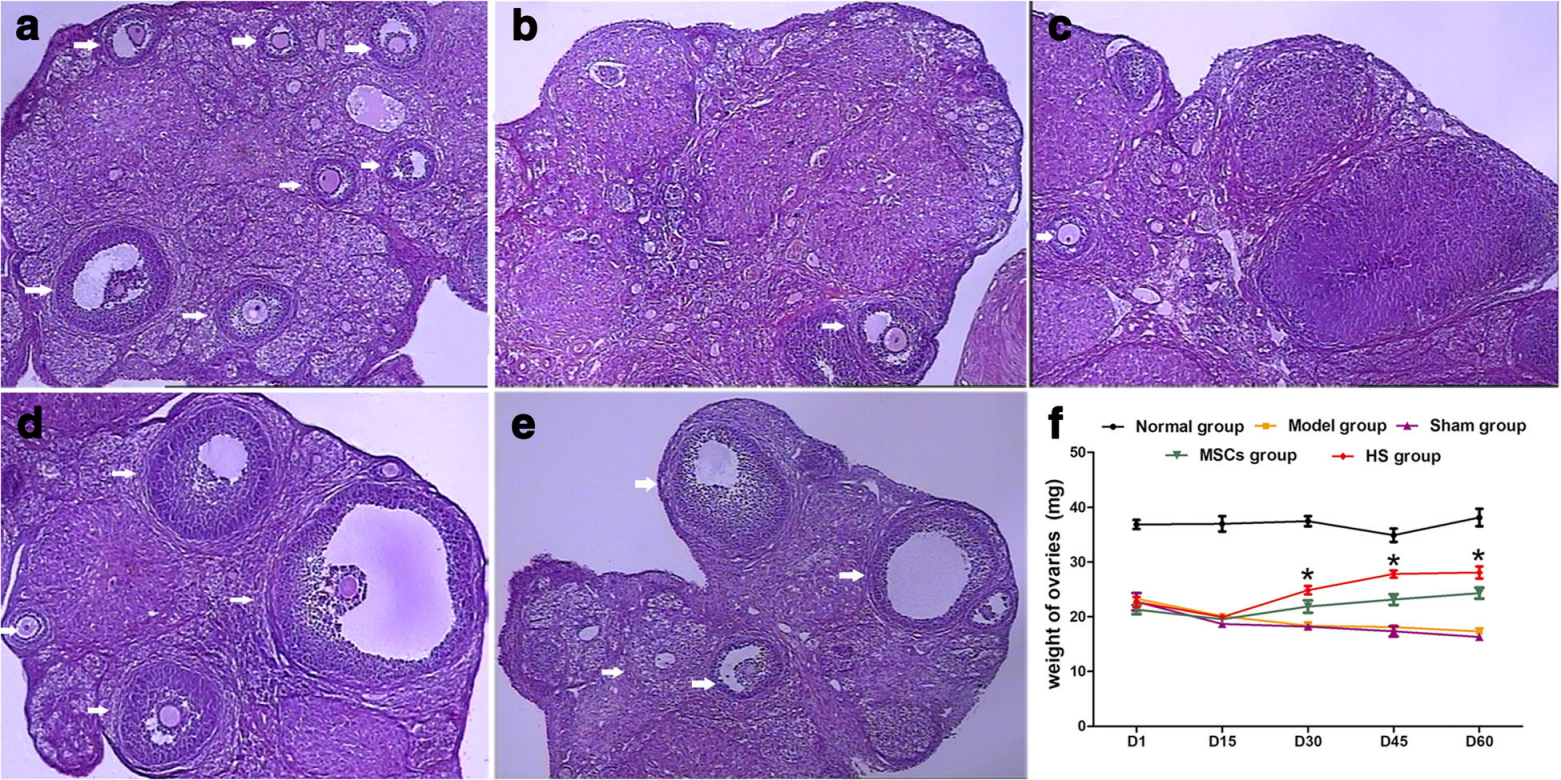
Ovarian structure and ovarian weight in each group. **a** Normal group (×50). **b** Model group (×50). **c** Sham group (×50). **d** MSCs group (×50). **e** HS group (×50). Arrows indicate follicles in ovary. **f** Ovarian weight of each group. *P < 0.05, compared with MSCs group. D day, HS heat shock, MSC mesenchymal stem cell

Rats in the normal group had normal appearance of ovaries, which were red in color with several white spot-like protuberances on the surface. At day 15 after CTX injection, the ovaries shrank in size in the model group, sham group, MSCs group and HS group; the surface of the ovaries was pale with reduced spot-like protuberances. At day 30, day 45 and day 60 after CTX injection, there were no apparent changes in the appearance of ovaries in the model group and sham group. In contrast, the ovaries increased in size at day 30, day 45 and day 60 after CTX injection in the MSCs group and HS group, with more spot-like protuberances, especially in the HS group. After HE staining, follicles at different developmental stages and several corpora lutea were observed in the ovaries of the normal group. Inside the follicles there were several layers of GCs. After CTX injection, the follicle count and corpora lutea count decreased. Ovarian angiogenesis, fibroplasia, vascular wall thickening and hyaline degeneration were observed. At day 45 and day 60 after CTX injection, the follicle count of the MSCs group and HS group increased compared to the model group and sham group (Fig. 5a–e).

The ovaries were weighed. At day 15 after injection, the ovary weight of the normal group was 36.99 ± 3.15 mg, which was higher compared to the model group (20.11 ± 0.44 mg), sham group (18.66 ± 1.47 mg), MSCs group (19.53 ± 0.79 mg) and HS group (19.93 ± 0.90 mg). However, there was no significant difference among the last four groups. At day 30, day 45 and day 60 post injection, the ovary weight increased in the MSCs group and HS group compared to the model group and sham group. The ovary weight gain was more apparent in the HS group and the weight was higher than that of the MSCs group (Fig. 5f).

**Fig. 6:**
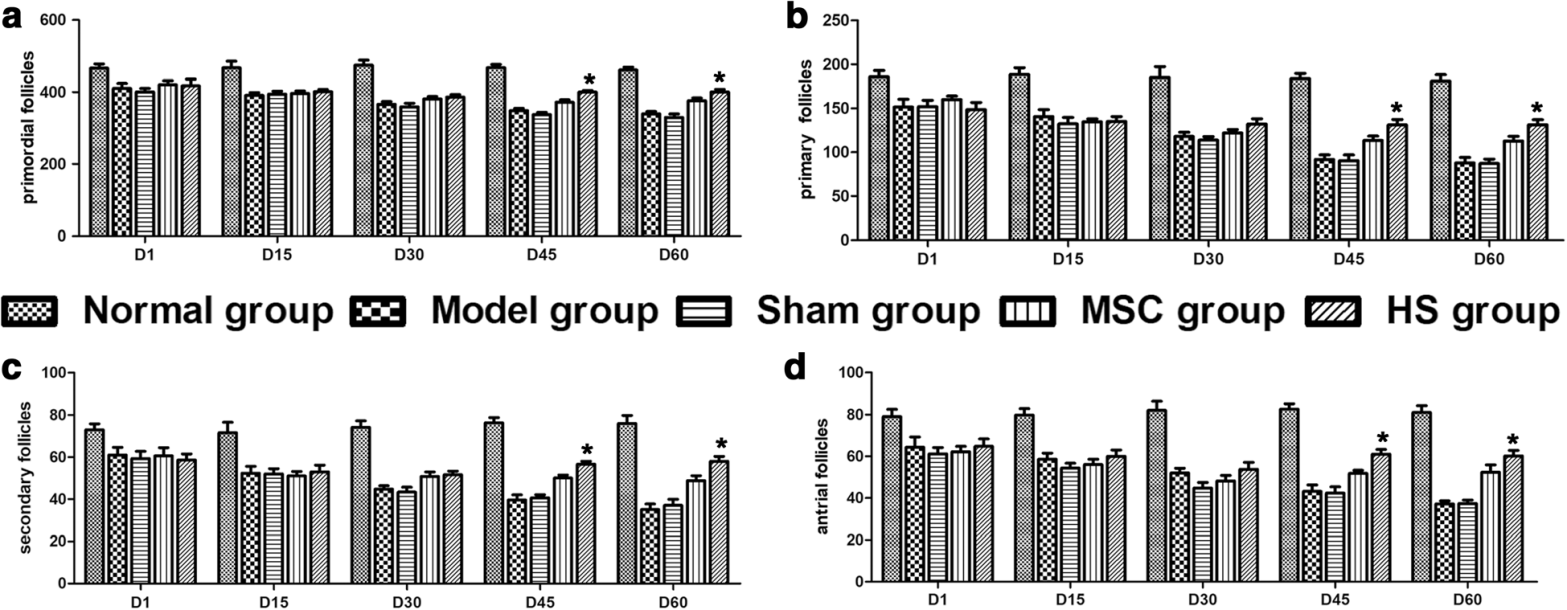
Amount of follicles at different stages in each group. **a** Primordial follicles. **b** Primary follicles. **c** Secondary follicles. **d** Antral follicles. *P < 0.05, compared with MSCs group. D day, HS heat shock, MSC mesenchymal stem cell

Follicles at different developmental stages were counted. At day 15 and day 30 post injection, the counts of follicles at different developmental stages were much higher in the normal group compared to the model group, sham group, MSCs group and HS group. There was no significant difference in the last four groups. At day 45 and day 60, the counts of follicles at different developmental stages further decreased in the model group and sham group. The counts of follicles at different developmental stages were higher in the MSCs group and HS group compared to the model group; and they were higher in the HS group than in the MSCs group. However, follicle counts in the HS group were still lower than those of the normal group (Fig. 6).

### Vaginal smears

The estrous cycle was normal in the normal group. In the model group, sham group, MSCs group and HS group, the estrous cycle was delayed, absent or persisted. There were six rats and eight rats in MSCs group and HS group which restored the normal estrous cycle at 16–30 days after the transplantation. There were three rats and five rats in these groups restoring a normal estrous cycle at 31–45 days after the transplantation. There were two rats and three rats restoring a normal estrous cycle at 46–60 days after the transplantation. However, there was no statistical difference in the MSCs group and HS group. The estrous cycle disturbance persisted in the model group and sham group (Table 1).

**Table 1:** Number of rats with a normal estrous cycle in each group

Group	1–15 days	16–30 days	31–45 days	46–60 days
Normal	20	15	10	5
Model	0	0	0	0
Sham	0	0	0	0
MSCs	0^*^	6^*^	3^*^	2^*,†^
HS	0^*,**^	8^*,**^	5^**,†^	3^**,†^

*HS* heat shock, *MSC* mesenchymal stem cell

^*^P < 0.0045 vs normal group

^**^P > 0.0045 vs MSCs group

^†^*P* > 0.0045 vs normal group

### Changes in sex hormone levels among the groups

**Fig. 7:**
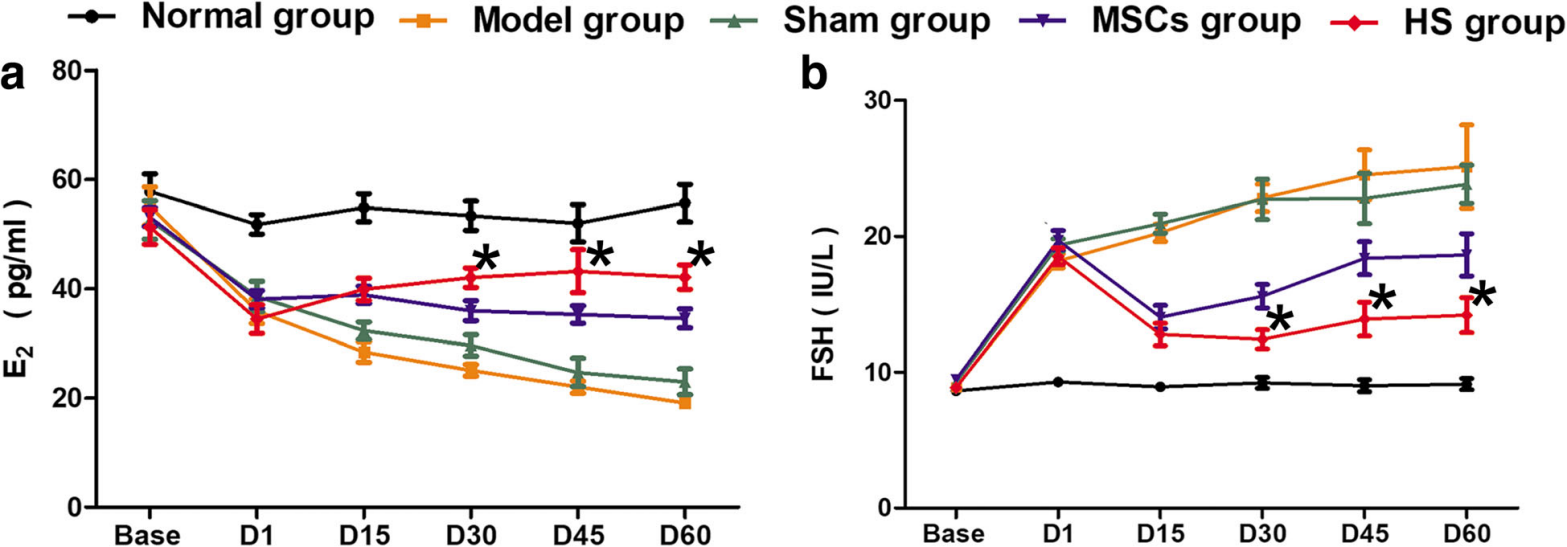
Sex hormone levels in each group. **a** Estradiol (E_2_). **b** Follicle stimulating hormone (FSH). *P < 0.05,compared with MSCs group. D day, HS heat shock, MSC mesenchymal stem cell

There was no significant difference in basic E_2_ and FSH levels between the groups (*F*_E2_ = 0.671, *P*_E2_ = 0.614; *F*_FSH_ = 1.773, *P*_FSH_ = 0.139). At day 1 post transplantation, the E_2_ levels of the model group, sham group, MSCs group and HS group were much lower compared to the normal group, while FSH levels were significantly increased compared to the normal group. One-way ANOVA indicated significant difference between the groups (*F*_E2_ = 9.419, *P*_E2_ = 0.000; *F*_FSH_ = 64.122, *P*_FSH_ = 0.000). However, pairwise comparisons of E_2_ and FSH levels among the four groups indicated no significant difference (*P* > 0.05). At day 30, day 45 and day 60 post injection, there were differences in sex hormone levels between the groups. They were maintained at baseline levels in the normal group; E_2_ levels decreased continuously in the model group, while FSH levels increased continuously. The sex hormone levels tended to stabilize in the HS group, and the difference was of statistical significance compared to the MSCs group; however, they were still lower than those of the normal group (Fig. 7).

### Apoptosis of rat GCs

**Fig. 8:**
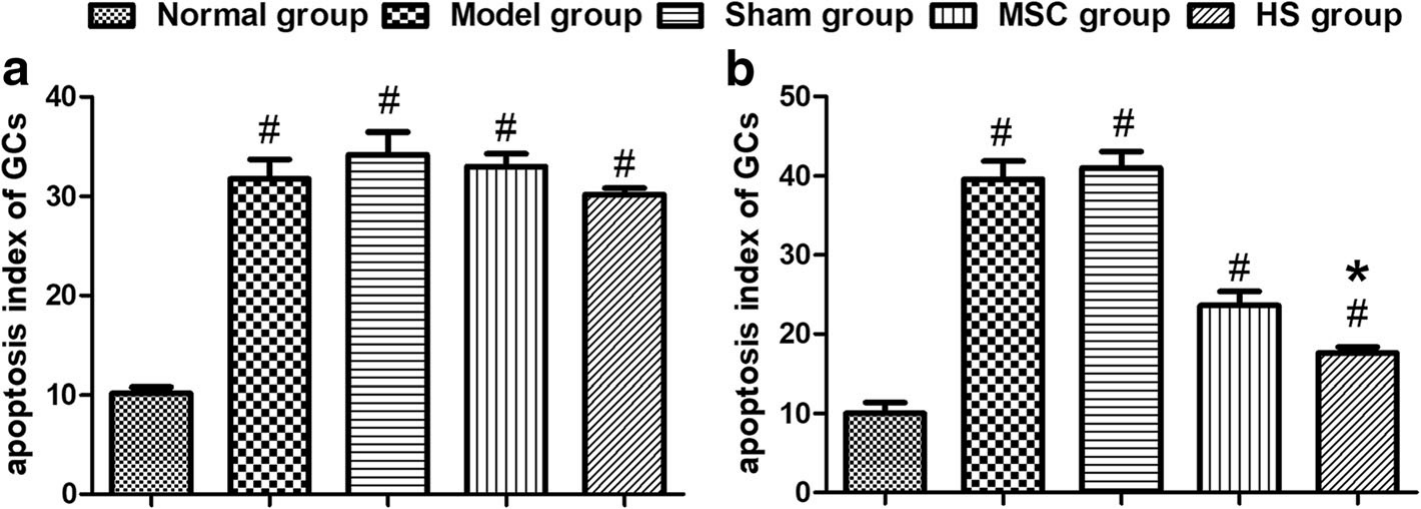
Apoptosis of ovarian granulosa cells in each group. Apoptosis index of ovarian granulosa cells at 1 day (**a**) and 60 days (**b**) after cell transplantation. #*P* < 0.05, compared with normal group; *P < 0.05, compared with MSCs group. GC granulosa cell, HS heat shock, MSC mesenchymal stem cell

At day 15 post injection, the apoptosis rates of GCs differed significantly among the five groups. The apoptosis rate of the normal group was 8.80% ± 2.39%, which was lower than that of the model group (35.80% ± 2.59%), sham group (37.80% ± 1.79%), MSC group (22.40% ± 3.36%) and HS group (18.20% ± 2.68%). Among the last four groups, the apoptosis rate of the HS group was lower than that of the model group, sham group and MSC group. At day 30, day 45 and day 60 post injection, the apoptosis rate of GC further decreased in the HS group; it was significantly lower compared to the model group, sham group and MSC group, and was also higher compared to the normal control group (Fig. 8).

## Discussion

Along with the extensive application of chemotherapy to cancer and autoimmune diseases, patient survival has been greatly improved. However, new problems such as chemotherapy-induced POF have emerged. Existing treatments for chemotherapy-induced POF include hormone replacement therapy [6], ovary cryopreservation and transplantation [7], in-vitro activation [8] and in-vitro fertilization–embryo transfer. However, none of them is a radical treatment.

Stem cells can repair the damaged organ structure and function [9, 10], thereby providing an alternative treatment for chemotherapy-induced POF. Stem cells may differentiate into damaged cells, and also secrete a variety of cytokines including vascular endothelial growth factor (VEGF), insulin-like growth factor-1 (IGF-1) and hepatocyte growth factor (HGF) to prevent cellular apoptosis [4, 11]. It has been reported that the survival of transplanted cells is usually low and that apoptosis and necrosis of the transplanted cells usually occur within 4 days after transplantation [12]. How to improve the survival and vitality of the transplanted stem cells remains a major challenge [13].

Heat shock (HS) is one of the major stress sources, which causes adverse impact on organisms and cells due to an abnormally high ambient temperature. HS can also be a pretreatment for stem cells prior to transplantation, which can increase the survival and reduce the apoptosis of the stem cells [14]. Cardiomyocytes receiving HS pretreatment at 43 °C for 30 min showed less glucose consumption and death caused by virus-activated fas-associated death pathway [12]. MSCs, after HS pretreatment of moderate intensity, displayed higher survival and proliferative capacity, and aging was delayed [15]. As for the optimal duration of HS pretreatment, no consensus has been reached because the experimented cells varied from one study to another. In our experiment, rate bone marrow-derived MSCs were used. It was found that HS pretreatment at 42 °C for 1 h significantly reduced the apoptosis of MSCs, which was consistent with Moloney et al. [16]. We also found that the anti-apoptotic effect of HS pretreatment persisted until 120 h post pretreatment, which corresponded to the peak apoptosis stage after stem cell transplantation. Therefore, HS pretreatment of MSCs can help improve survival and treatment efficacy. However, HS pretreatment of high intensity increased the apoptosis rate of MSCs until 48 h post pretreatment, when the apoptosis rate began to decrease. This is because HS itself is a stimulus that causes damage to the cells in the short term. But as the cells recover from the damage over time, more heat shock proteins (HSPs) are generated and the anti-apoptotic capacity of the cells is enhanced accordingly. In one study, after HS pretreatment of MSCs at 42 °C, the expression levels of HSP27 and HSP70 were upregulated significantly [16]. This means HSPs were upregulated for some time after HS pretreatment at certain intensity. HSPs as molecular chaperones play an important role under stress by promoting accurate protein folding and accelerating the recovery of normal protein synthesis [17], thereby increasing the anti-apoptotic capacity of the cells. The growth features of the pretreated cells were further studied. Cell cycle analysis indicated that after HS pretreatment a higher percentage of MSCs were arrested in the S phase compared to the nonpretreated MSCs. According to CCK-8 assay, the proliferation of MSCs was accelerated at 48 h post pretreatment. This indicated the promoting effect of HS pretreatment for 1 h on the proliferation of MSCs. Following HS pretreatment, the expression of silent information regulator 1 (SIRT-1) was upregulated [15], cell apoptosis and aging were inhibited [18] and cell proliferation was promoted [19]. In the meantime, HSP90 was generated in a greater quantity after HS pretreatment, which was conducive to the proliferation and migration of the target cells [20].

We further studied the effect of HS pretreatment on the apoptosis of MSCs in the local microenvironment under chemotherapy, which was mimicked by adding PM into the culture medium. After HS pretreatment, apoptosis of MSCs was reduced, while tolerance to chemotherapy was strengthened. Coculture of the HS-pretreated MSCs with GCs also improved the anti-apoptotic capacity of GCs. The anti-apoptotic effect of HS pretreatment is closely associated with HSPs. An upregulation of HSPs results in heat resistance of the cells for some time and temporary resistance to subsequent stress. HS pretreatment can induce the generation of heat shock transcription factor (HSF1), which activates specific signaling pathway (e.g., HSF1/miR-34a/HSP70) to produce a variety of HSPs [21]. HSPs are involved in the blockage of several apoptotic pathways. For example, HSP27 and HSP90 can bind to Apaf-1, which blocks the formation of apoptosomes and the mitochondrial apoptotic pathway. HSP70 interacts with apoptosis inducing factor (AIF) to block the caspase-mediated apoptotic pathway [22]. Moreover, autophagy is also involved in HS, so appropriate autophagy is important to cell survival. In the H_2_O_2_-induced cellular apoptosis model, activation of the p38MAPK/mTOR pathway may be the mechanism of HS increasing the survival of MSCs [23]. Herberg et al. [24] reported that the stromal cell-derived factor 1 (SDF-1)/CXC chemokine receptor 4 (CXCR4) signaling axis was crucial to the prolife ration and survival of MSCs by enhancing the autophagy under oxidative stress. Coculture between MSCs and GCs resulted in suppressed apoptosis of GCs via the paracrine pathway [3]. After HS pretreatment, the survival of MSCs was enhanced and their ability to inhibit GCs was also improved. HSP90 can improve the migratory capacity of MSCs via the PI3K/AKT and ERK pathways, thus promoting migration of the stem cells to the site of injury [25]. It is able to repair the damaged tissues and cells by secreting a variety of cytokines and inhibiting apoptosis of the target cells.

Many reports focused on the effect of HS pretreatment on improving the therapeutic efficiency of stem cell transplantation in animal experiments. In the rat model of myocardial injury, HS pretreatment was performed before stem cell transplantation, which could reduce apoptosis of the transplanted cells by about 54% [12]. Transplantation of HS-pretreated sca-1^+^ cells to the rat model of myocardial infarction significantly reduced cell apoptosis and fibrosis, thereby improving the overall cardiac function of the ischemic myocardium [21]. In the rabbit model of liver ischemia/reperfusion, transplantation of HS-pretreated MSCs significantly reduced cell apoptosis. In addition, there was a reduction in serum aminotransferase and Suzuki scores with histopathological improvement and more PCNA-positive cells. This indicated an improvement in the efficacy of stem cell transplantation for liver damage [23]. In our study, the estrous cycle was restored in some rats after transplanting the HS-pretreated MSCs to the rat ovaries damaged by chemotherapy. At day 45 after cell transplantation, the counts of follicles belonging to different developmental stages were higher than those of the MSCs group. At day 30 after transplantation, the serum E_2_ level was higher than that of the MSC group, while the FSH concentration was lower than that of the MSC group. It was hence indicated that HS pretreatment of MSCs was better in improving the damaged ovarian structure and endocrine function after chemotherapy. In a word, this treatment displayed greater repair effect on chemotherapy-induced POF. We detected the apoptosis of GCs and found a reduction in the apoptosis rate after HS pretreatment, which was consistent with in-vitro experiment. GCs are the largest cell group comprising the follicles and the major sources of estrogen and progesterone. They play an important role in maintaining normal structure and function of follicles. The apoptosis of GCs is closely related to follicular atresia [26]. As the apoptosis of GCs decreases, so does follicular atresia. As a result, ovarian structure and endocrine function are restored. Hence, the ability of HS pretreatment in enhancing the repair effect of MSCs is associated with the inhibited apoptosis of GCs. Based on the animal experiment, heat-shocked MSCs have the potential to become a practical and reliable clinical treatment for POF in the future.

## Conclusions

The present study demonstrated that: HS pretreatment at 42 °C for 1 h inhibited apoptosis and promoted proliferation of MSCs; and after HS pretreatment, apoptosis of MSCs in the local microenvironment under chemotherapy was reduced. Also, HS preconditioned MSCs can further inhibit the chemotherapy-induced apoptosis of GCs when they are cocultured: transplantation of MSCs pretreated by HS further improved ovarian structure and endocrine function under chemotherapy. The enhanced repair effect was directly associated with the reduced apoptosis of GCs.

## Abbreviations

E_2_: Estradiol
FSH: Follicle stimulating hormone
GC: Granulosa cell
HS: Heat shock
MSC: Mesenchymal stem cell
POF: Premature ovarian failure
